# Percutaneous Transluminal Angioplasty and Stenting for Symptomatic Intracranial Stenosis After SAMMPRIS: Patient Selection and Clinical Outcomes

**DOI:** 10.3390/jcm15020633

**Published:** 2026-01-13

**Authors:** Leonhard Mann, Patrick Felix Samp, Jan Hendrik Schaefer, Elke Hattingen, Joachim Berkefeld, Dimah Hasan, Fee C. Keil

**Affiliations:** 1 Institute of Neuroradiology, University Hospital Frankfurt, Goethe-University, 60596 Frankfurt am Main, Germany; 2 Department of Neurology, University Hospital Frankfurt, Goethe-University, 60596 Frankfurt am Main, Germany; 3 Department of Diagnostic and Interventional Neuroradiology, Hospital Aachen, RWTH University, 52062 Aachen, Germany

**Keywords:** intracranial stenosis, angioplasty, stenting, stroke

## Abstract

**Background/Objectives:** After the negative results of the SAMMPRIS trial, the indication for endovascular treatment of atherosclerotic intracranial artery stenosis (ICAS) was widely restricted. It was the aim of our study to report whether intracranial arterial percutaneous transluminal angioplasty and stenting (PTAS) as ultima ratio therapy is still effective and safe enough. **Methods:** Between February 2011 and June 2019, 63 consecutive patients with and without emergent large vessel occlusion (ELVO) who received PTAS for symptomatic ICAS in the anterior or vertebrobasilar circulation were included in our study. **Results:** A total of 32 patients had ELVO. In the remaining 31 patients, a known ICAS was treated with PTAS either because of recurrent stroke despite aggressive medical therapy with dual antiplatelet inhibition (*n* = 24) or due to progressive hemodynamic ischemia (*n* = 7). Stenting was successful in all 63 cases. Successful reperfusion was achieved in 94% of ELVO patients. Complications with new neurologic deficits, including dissection, subarachnoid hemorrhage, intracerebral hemorrhage (PH2), and stent thrombosis, were seen in five ELVO patients (16%). At discharge, neurological status improved in 16 patients (50%) and deteriorated in 7 patients (22%). In-hospital mortality happened in 5 of 32 ELVO cases (16%), and all of them had lesions in the vertebrobasilar circulation. Regarding non-ELVO cases, two patients (6%) developed new neurologic deficits due to perforator strokes. There was no in-hospital mortality in this group. **Conclusions:** Even in unfavorable situations with acute atherothrombotic occlusions or recurrent strokes under aggressive medical therapy of known ICAS, PTAS remains a treatment option with reasonable effectiveness. This should be balanced against other treatment options, taking into account the complication rate, which is not negligible.

## 1. Introduction

In western countries, atherosclerotic intracranial artery stenosis (ICAS) is a relatively rare but important cause of ischemic stroke. It is often a therapeutic challenge because of its high risk for recurrent stroke despite medical therapy and/or interventional stenting [1,2,3,4]. The SAMMPRIS trial (Stenting and Aggressive Medical Management for Preventing Recurrent Stroke in Intracranial Stenosis) addressed this issue and randomized patients with high-grade ICAS to either aggressive medical therapy alone or aggressive medical therapy with intracranial arterial percutaneous transluminal angioplasty and stenting (PTAS) [2]. It was found that aggressive medical therapy alone was superior to the regime that included PTAS, mainly due to high periinterventional complication rates in the PTAS group [2,3,4,5].

The results of the SAMMPRIS trial led to considerable changes in treatment strategies for symptomatic ICAS patients towards a clear plan of optimized medical therapy, limiting the indications for interventional treatment. According to current guidelines, PTAS may be considered only in cases with new symptoms and infarcts despite medical treatment with dual antiplatelet therapy (DAPT) or as a form of rescue in cases with acute stroke due to emergent large vessel occlusion (ELVO) on the basis of underlying ICAS [6]. In most instances, ELVO patients with atherothrombotic occlusions are identified during failed thrombectomy procedures, ending up with emergency PTAS as ultima ratio treatment with a need for sufficient antiplatelet therapy. In this case, the associated risks of stent thrombosis on one side and intracranial hemorrhage on the other side are difficult to control and may contribute to increased complication rates [7].

For the other group of patients with failure of medical therapy, it is not clear whether PTAS as ultima ratio therapy is a source of further complications or if it would be helpful in avoiding permanent disability [8,9,10].

It is a common impression among neurointerventionalists that the rate of unfavorable outcomes is high in both patient groups (ELVO and non-ELVO). However, literature data addressing the real-life outcomes of PTAS after SAMMPRIS are scarce and heterogeneous regarding inclusion criteria and outcome parameters [11,12,13,14,15,16,17,18].

This is why it was the aim of our study to review all PTAS procedures in the latter two mentioned groups of indication in the period after publication of the SAMMPRIS trial from 2011–2019 in order to evaluate the effectiveness and safety of PTAS regarding angiographic and in-hospital clinical outcomes.

## 2. Materials and Methods

### 2.1. Patient and Data Acquisition

This retrospective study was approved by our institutional ethics committee Nr, 430/18. For the analysis, we searched databases for patients who received PTAS in our institution between February 2011 and June 2019.

Inclusion criteria were age 18 to 99 years, intracranial stenting performed at our institution during the study period, and the presence of high-grade intracranial stenosis defined as 70 to 99 percent stenosis according to the WASID criteria in a major intracranial artery, specifically the internal carotid artery, the M1 segment of the middle cerebral artery, or the basilar artery.

Patients who underwent rescue stenting during acute endovascular stroke treatment for an acute symptomatic large-vessel occlusion were assigned to the ELVO group.

In contrast, patients treated outside the acute setting for symptomatic intracranial stenosis—including those with recurrent or progressive infarctions or transient ischemic attacks within the vascular territory of the affected intracranial artery despite dual antiplatelet therapy—or those treated because of hemodynamic compromise were assigned to the non-ELVO group. Non-ELVO patients were required to have had a qualifying ischemic event no more than 90 days prior to the procedure.

### 2.2. Study Population 

**Figure 1:**
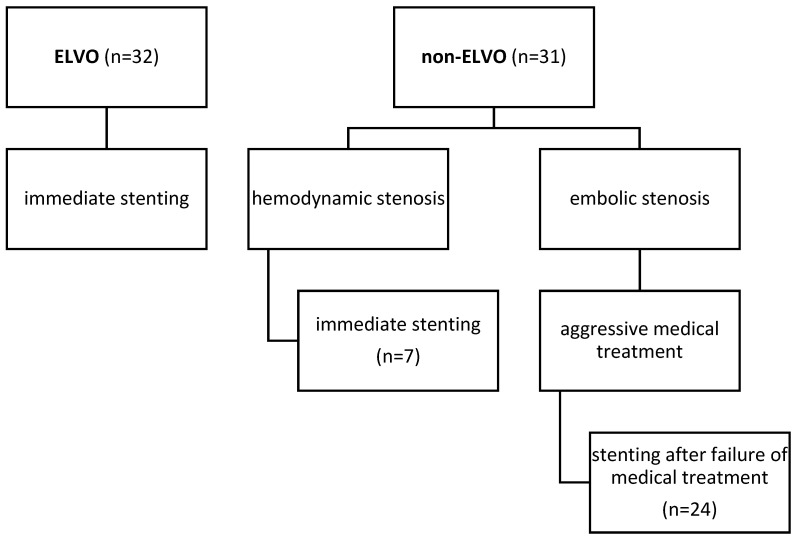
Chart illustrating causes for stenting in our cohort.

We excluded all cases with intracranial stents that were not placed for ICAS, such as aneurysm or arterial dissection treatment. This left 63 consecutive patients to be included. The selection process is shown in Figure 1. We assessed the clinical and radiological data of these patients. All imaging data were reviewed by two independent neuroradiologists, and a reference standard was established for statistical analysis. Due to the retrospective study design and the use of routine clinical documentation and imaging, the neuroradiologists were not blinded to treatment group or clinical outcome.

### 2.3. Procedures

All procedures were performed under general anesthesia in a biplane angiography suite (Axiom artis zee, Siemens Healthineers, Erlangen, Germany). Stenosis degree and the diameters of the adjacent normal arterial segments were determined using digital subtraction angiography (DSA) images and volume rendering reconstruction of an additional 3D rotational angiography. Stenosis degree was measured according to WASID (Warfarin–Aspirin Symptomatic Intracranial Disease trial) criteria.

### 2.4. Non-ELVO Case

**Figure 2:**
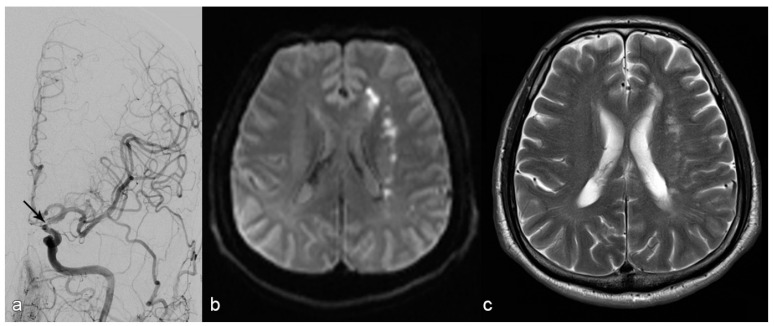
Patient with stroke due to stenosis in the terminal segment of the left internal carotid artery ((**a**): arrow). MR diffusion weighted imaging upon admission (**b**) shows subacute infarction in the white matter of the left centrum ovale with a hemodynamic pattern and without embolic cortical infarction. The decision was made to place a stent, which succeeded; MRI upon discharge showed no additional infractions (**c**).

For non-ELVO cases (example given in Figure 2), we largely deployed balloon-expandable stents. The diameter of balloon-expandable stents was at least 0.25 mm smaller than the normal vessel diameter to avoid over-dilatation. However, self-expanding stents were used if the target vessel diameter was below 2.5 mm or if the stenosis was not accessible with balloon-expandable stents. In cases of self-expanding stent-placement, stenoses were pre-dilated with an undersized balloon.

### 2.5. ELVO Case

**Figure 3:**
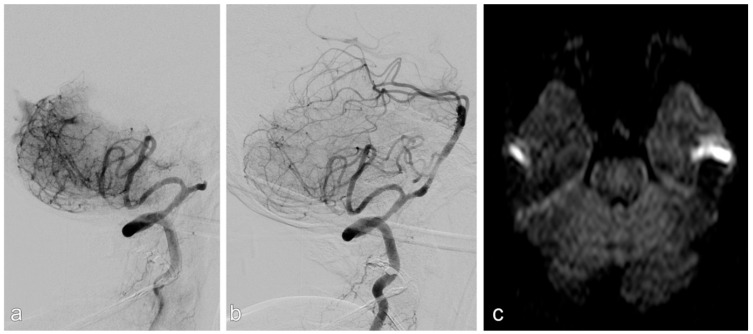
Patient with acute stroke due to acute occlusion of the basilar artery (**a**). After mechanical thrombectomy, there was a high-grade stenosis, which was stented successfully (**b**). There was no infarction on MRI upon discharge (**c**).

In ELVO cases (example given in Figure 3), mechanical thrombectomy was always performed first using either a non-detachable stent retriever and/or an aspiration catheter. If the angiographic result was insufficient, including persistent occlusion, re-occlusion attributable to an underlying stenosis, or a residual high-grade stenosis, rescue treatment with a self-expanding stent (Solitaire AB, Medtronic, Minneapolis, MN, USA) was performed. If necessary, stenoses were dilated pre and/or after stent placement with an undersized balloon. In some cases, balloon-expandable stents were used for this indication, if feasible.

### 2.6. Antiplatelet Management

All non-ELVO patients were treated with dual antiplatelet therapy before PTAS. Patients undergoing elective PTAS while already on maintenance DAPT received aspirin (Bayer AG, Leverkusen, Germany) 100 mg and clopidogrel (Sanofi, Paris, France) 75 mg, and antiplatelet medication was not modified for the stenting procedure. In patients treated for recurrent ischemic events despite medical therapy, “DAPT failure” was defined as at least five days of continuous aspirin 100 mg plus clopidogrel 75 mg prior to PTAS. In patients treated primarily for hemodynamic compromise, DAPT was initiated with a loading regimen of clopidogrel 600 mg and aspirin 100 mg administered orally on the day before the procedure. All non-ELVO patients received intravenous heparin (Heparin sodium; Braun, Melsungen, Germany) at 70 IU/kg body weight.

In emergent ELVO cases, intravenous heparin at 70 IU/kg and a tirofiban (Aggrastat; Medicure International, Winnipeg, MB, Canada) bolus of 0.4 µg/kg were administered intra-procedurally before stent deployment, followed by a tirofiban infusion of 1 µg/kg/h. During the intervention, patients additionally received a loading dose of clopidogrel 600 mg orally and aspirin 250 mg intravenously. The tirofiban infusion was discontinued four hours after the procedure.

Pre-procedural platelet function testing was performed using point-of-care assays, including APA-ACT to assess aspirin effect and a P2Y12 assay to assess clopidogrel responsiveness. Tirofiban was not used routinely in elective cases and was reserved for emergent ELVO cases as bridging therapy when clinically indicated.

### 2.7. Procedural Timing

ELVO patients underwent immediate intervention. In non-ELVO patients with hemodynamically relevant stenoses, stenting was performed within 24 h, whereas patients who became symptomatic during the ongoing 90-day DAPT period were treated within that treatment window and after at least five days of therapy. Time to procedure was not collected for this study.

### 2.8. Outcome Parameters

Because of the different disease characteristics of these two groups (ELVO and non-ELVO), the outcome parameters were evaluated separately.

### 2.9. Outcome Parameters in ELVO Patients

Primary outcome parameters were technical success and reperfusion success as well as the occurrence of adverse events.

Stenting was defined as successful (technical success) if a residual stenosis of ≤50% was achieved. Reperfusion was defined as successful (reperfusion success) if complete or almost complete reperfusion (mTICI ≥ 2b) was achieved. Adverse events included intervention-related complications such as (1) intracranial dissections, (2) acute and subacute stent thrombosis, (3) subarachnoid hemorrhage (SAH), and (4) symptomatic intracranial hemorrhage (parenchymal hematoma type 2 “PH-2” according to the European Cooperative Acute Stroke Study “ECASS II” classification).

Secondary outcome parameters were early clinical neurological outcomes during hospital stay and at discharge. Neurological outcome was assessed using the National Institutes of Health stroke scale (NIHSS), with early improvement (and deterioration) being defined as decrease (and increase) from the baseline NIHSS immediately pre-procedure by at least 4 points to the NIHSS at discharge, respectively. Furthermore, clinical outcome at discharge was assessed using the modified Rankin scale (mRS), with good clinical outcome being defined as mRS = 0–2.

To address the possible different natural courses of processes in anterior and vertebrobasilar circulations, we assessed all primary and secondary outcome parameters separately for both circulations [19].

### 2.10. Outcome Parameters in Non-ELVO Patients

Primary outcome parameters were technical success as well as the occurrence of adverse events (defined previously).

The secondary outcome parameter was newly occurring stroke post-procedure. In this group, the neurological situation was compared to the asymptomatic state before the procedure during clinical stability.

We also assessed all primary and secondary outcome parameters separately for both circulations.

### 2.11. Statistics

Parametric variables are indicated as mean ± standard deviation (SD), and non-parametric variables are indicated using median and interquartile range (IQR). For comparison of these variables, we used Student’s *T* test or the Mann–Whitney *U* test after testing for normal distribution with a Shapiro–Wilk test. Nominal variables were tested with Fisher’s exact tests and χ^2^ tests depending on sample size. *p* values under the α-level of 0.05 were defined as significant. Multivariable analysis was performed with a binary logistic regression test indicating odds ratios (ORs) and 95% confidence intervals (CIs). All statistical analyses were performed with SPSS-25 software.

## 3. Results

### 3.1. Baseline Characteristics

The baseline characteristics of all patients can be found in Table 1. In summary, 32 of 63 patients (51%) had acute strokes with ELVO. In the remaining 31 patients (49%) (non-ELVO group), intracranial stenosis was treated with PTAS either because of recurrent stroke with infarctions despite aggressive medical therapy with DAPT (*n* = 24) or due to hemodynamic insufficiency with progressive symptoms and infarctions (*n* = 7).

Except for history of stroke and previous medication, there were no other significant differences between the two groups regarding demographic data and comorbidities (Table 1).

The distribution of anterior and vertebrobasilar circulation was also comparable (*p* = 0.319). Among patients with anterior circulation pathology, middle cerebral artery (MCA) stenosis was more frequent in the ELVO group (38% vs. 16%), whereas internal carotid artery (ICA) pathology was more frequent in the non-ELVO group (36% vs. 9%).

In ELVO cases, the diagnosis of an underlying stenosis was made after a median of 1 (interquartile range or IQR, 1–3) thrombectomy maneuver.

### 3.2. Outcome Parameters in the ELVO Group

Regarding technical success, stenting was technically successful in all cases (Table 1).

Successful reperfusion (mTICI ≥ 2b) was achieved in 94% of ELVO patients (Figure 3).

Intervention related complications and post-interventional imaging findings can be found in Table 2. The main complications are summarized as follows:

Intracranial dissections with compromised side branch patency occurred in 2 out of 32 patients. Both cases were in the vertebrobasilar circulation and occurred after balloon angioplasty. In one of these two cases, there was subsequent in-stent thrombosis, which was fatal. In the other case, there were embolic and perforator infarction, which led to worsening of the neurologic symptoms.

Acute and subacute stent thrombosis were observed in two cases, both of which occurred in the vertebrobasilar circulation and were symptomatic. One of these two patients had progressive infarction, which led to neurological worsening (mRS = 5); the other had stent thrombosis after dissection, which was fatal (as mentioned previously).

There was no SAH.

Symptomatic intracranial hemorrhage occurred in two ELVO patients, both in the anterior circulation. Only one of these two patients had intravenous thrombolysis therapy before the procedure. One patient recovered and improved neurologically (mRS = 2); the other one deteriorated (mRS = 4).

According to NIHSS, 50% of patients had neurological improvement upon discharge. Death within 30 days occurred in five cases (16%) in this group. Secondary outcome parameters regarding good clinical outcome at discharge (mRS = 0–2) were observed in 31% of ELVO cases. When only ELVO patients with stenosis in the anterior circulation were considered, good clinical outcomes were observed in 40% of the cases.

### 3.3. Outcome Parameters in the Non-ELVO Group

Stenting was also technically successful in all cases in this group. Regarding intervention-related complications, there were no intracranial dissections, acute or subacute in-stent thrombosis, SAH, or symptomatic intracranial hemorrhage.

Two patients in this group (6%) had new stroke after the procedure because of periinterventional perforator infarctions. Deaths within the first 30 days did not occur in the non-ELVO group.

There was one case of severe pneumonia with the necessity of prolonged post-interventional ventilation as a non-neurologic complication.

### 3.4. Outcome Pre- and Post-mRs in ELVO and Non-ELVO

**Figure 4:**
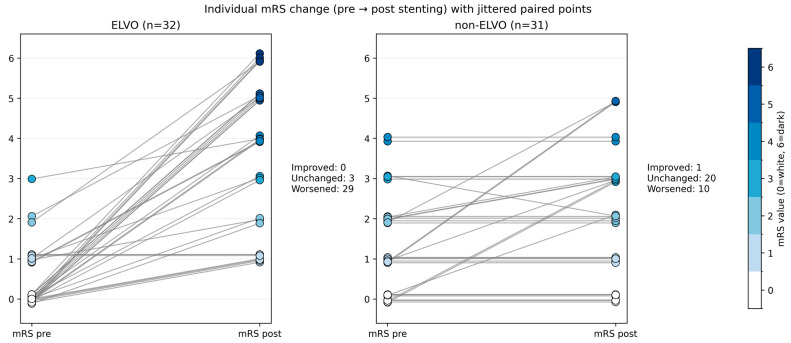
Individual modified Rankin scale (mRS) change before and after intracranial stenting in emergent ELVO and elective non-ELVO patients. Each line represents one patient, connecting the pre-event or pre-admission mRS (**left**) with the discharge mRS (**right**). Marker color encodes mRS severity, with mRS 0 shown in white and mRS 1–6 displayed in progressively darker blue shades. Lines are shown in medium gray to facilitate visual tracking of within-patient change.

The patients level of independence was assessed using the mRS immediately before the stenting and shortly before discharge. Differences between the ELVO and the non-ELVO group are shown in Figure 4.

### 3.5. Outcome NIHSS Change in ELVO and Non-ELVO

**Figure 5:**
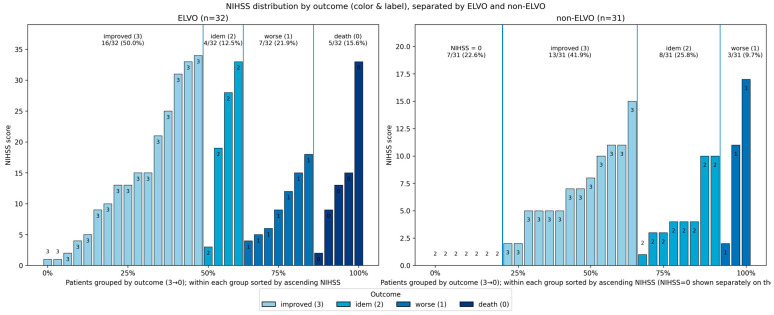
Plots demonstrating the distribution of baseline neurological deficit in ELVO and non-ELVO patients, stratified by early in-hospital outcome category. Patients are grouped according to their outcome class, with the height of each bar representing the individual NIHSS score and the color of each bar indicating outcome severity from improvement to death. Within each outcome block, patients are ordered by ascending NIHSS, and a separate block on the far left displays patients with NIHSS equal to zero. The proportions displayed above each block provide a summary of the share of patients within each outcome category for the respective cohort.

The NIHSS outcome illustrated in Figure 5 plots demonstrates the distribution of baseline neurological deficit in ELVO and non-ELVO patients, stratified by early in-hospital outcome category. Patients are grouped according to their outcome class, with the height of each bar representing the individual NIHSS score and the color of each bar indicating outcome severity from improvement to death. Within each outcome block, patients are ordered by ascending NIHSS, and a separate block on the far left displays patients with NIHSS equal to zero. The proportions displayed above each block provide a summary of the share of patients within each outcome category for the respective cohorts.

### 3.6. Comparison of Anterior and Vertebrobasilar Circulation

All fatal cases were ELVO cases in the vertebrobasilar circulation (*p* = 0.022).

Furthermore, all vessel dissections occurred in the vertebrobasilar circulation, and all intracranial hemorrhages happened in the anterior circulation. However, these two differences did not reach statistical significance.

## 4. Discussion

From a technical point of view, all stenting procedures were successful, with less than 50% residual stenosis and no differences between both groups. In the group with ELVO and emergent intervention in the anterior and vertebrobasilar circulation, we achieved good reperfusion results (mTICI ≥ 2b) in 94% of cases, which is higher than the cumulative reperfusion rate of 71% after thrombectomy of embolic stroke in anterior circulation without PTAS in the HERMES study [20]. This rate is comparable with other stenting trials in anterior cerebral circulation and is somewhat higher than the successful reperfusion rate of 80% reported by Baek et al. when varying rescue treatments for ICAS in anterior circulation were used (including balloon angioplasty, stenting, and intra-arterial glycoprotein IIb/IIIa inhibitor infusion) [21,22,23]. The periprocedural complication rate in our ELVO patients was significantly higher than in the more elective non-ELVO cases. Severe adverse events like symptomatic hemorrhage or stent thrombosis as well as mortality were seen only in ELVO patients.

However, these complication rates are comparable to data reported from thrombectomy trials without stenting [20,21].

The rates of hemorrhagic complications in our ELVO group were in accordance with results by Baek et al., who analyzed ELVO patients and found no added bleeding risk when additional endovascular therapy was used [21].

Furthermore, intracranial hemorrhage occurred in two patients (6%), placing our hemorrhagic complication rate within the lower and generally comparable range reported in major thrombectomy cohorts. For example, the HERMES individual patient meta-analysis reported a parenchymal hematoma type 2 rate of 5.1% and a symptomatic intracranial hemorrhage rate of 4.4%. In the TITAN pooled analysis by Gory et al., parenchymal hematoma occurred in 13.8%, indicating that our observed hemorrhage rate is at the lower end when compared with some large real-world tandem-lesion thrombectomy cohorts [20,24]. In accordance with the literature, our findings support that administration of intravenous thrombolysis was no risk factor for hemorrhage despite DAPT, implying that thrombolysis should not discourage from stenting if necessary [24]. However, to avoid potential hemorrhagic risks of aggressive DAPT, some authors have proposed using balloon angioplasty without stenting [25]. The limitation of this approach in terms of vessel patency is known [26]. In attempts to use angioplasty only, we frequently observed re-obstruction due to elastic recoil or dissection during the intervention. Due to the fact that intracranial stent thrombosis is in most instances a catastrophic event, we preferred adequate antiplatelet therapy with immediate action [27]. However, according to this data, it remains unclear whether antiplatelet therapy including the use of glycoprotein (GP) IIb-IIIa antagonists increases the rate of hemorrhagic complications.

Regarding vessel dissection and stent thrombosis, these are probably specific complications for procedures including angioplasty, with a relatively high rate of 9% in our sample. Plaque alteration and rupture after stent retriever maneuvers or balloon dilation may play a role in the development of a highly thrombogenic surface. To the best of our knowledge, data about the rates of these complications among ELVO patients undergoing emergent PTAS do not exist. A favorable clinical outcome (mRS = 0–2) upon discharge was achieved in 31% of patients in the ELVO group. These numbers appear relatively low given that good clinical outcome rates in ELVO patients with embolic occlusion are higher in some thrombectomy trials (for example, 71% in EXTEND-IA [28] and 60% in SWIFT-PRIME [29]).

However, a closer look at our data reveals that this is mainly because we included multimorbid patients with unfavorable neurological baselines, high-grade stenoses, and stenoses in the vertebrobasilar circulation (Table 1 and Table 2), which often have a worse clinical course [19]. In fact, when only ELVO patients with stenosis in the anterior circulation are considered, our good clinical rate reaches 40% without mortality cases which occurred only in the vertebrobasilar circulation. This is comparable to the 46% indicated by Baek et al. in the anterior circulation [21]. Approaching clinical outcome more pragmatically and taking into account patients’ individual baseline, our results are more favorable: 50% of ELVO-patients with PTAS improved neurologically. This is comparable to results in the HERMES study for embolic strokes. Thus, undertaking endovascular recanalization including acute PTAS seems worthwhile in this group of patients. In non-ELVO patients, the procedure was again technically successful in all cases. However, at 6%, the rate of in-hospital stroke and/or neurological deterioration was significantly lower in comparison to the ELVO cases (22%) (*p* = 0.041). There were no death cases among the non-ELVO patients compared to an in-hospital mortality rate of 16% in the ELVO group (*p* = 0.022). Importantly, no vessel dissection or stent thrombosis was seen, which supports the consideration that these complications, which were seen in the ELVO group, could be not directly related to the stent implantation but more to the thrombectomy itself or to preceding balloon angioplasty. Emergent antithrombotic therapy may also play a role in this regard. We observed two perforator strokes with new deficits which are typical PTAS complications in the posterior circulation [30].

Another patient with multimorbidity deteriorated due to hospital-acquired pneumonia. The rates of strokes (6%) and deaths (0%) were lower than the periprocedural complication rate in the SAMMPRIS trial (16.3%) and interestingly comparable with the results reported by Meyer L et al., who found a periprocedural rate of stroke and death of 6.5% [31]. It is hypothesized that a number of factors may explain the lower periprocedural complication rate observed in the non-ELVO cohort in comparison to the rate reported in the SAMMPRIS cohort. Firstly, it is important to note that our non-ELVO interventions were performed in an elective clinically stable setting rather than during acute stroke care. This approach circumvents the concomitant procedural intricacies and hazards associated with thrombectomy, thereby mitigating the probability of intervention in a vessel segment that has already sustained acute injury or destabilization due to thrombus manipulation and endothelial trauma. Secondly, all non-ELVO patients were treated under established dual antiplatelet therapy, which may have mitigated acute thrombotic and embolic complications, such as stent thrombosis and distal embolization. Our ischemic complication rate was comparable to rates reported by Aghaebrahim et al. in their analysis of patients with ICAS, who received PTAS with balloon-expandable and self-expanding stents after aggressive medical management [26]. A reduced incidence of thrombotic events may also result in a decreased requirement for aggressive antithrombotic interventions, which could in turn contribute to a reduction in the burden of hemorrhagic complications.

Thirdly, in contrast to SAMMPRIS, our approach enabled individual selection of devices based on the morphology of lesions and the anatomy of vessels [5]. In tortuous segments, the deployment of more flexible self-expanding stents was chosen to optimize navigability and wall apposition. In contrast, in high-grade stenoses where radial force was considered critical, balloon-mounted stents with more precise deployability were utilized. This bespoke strategy may have enhanced technical performance and diminished procedure-related adverse events in comparison with the fixed-device trial protocols.

Mohammaden et al. also demonstrated a comparable periprocedural stroke rate of 5.6% using BMS in a multicenter study [32]. A more recent review, discusses that the advantages of the high radial force of BMS only seem to be offset by the disadvantages of more difficult navigability through vascular curves at lengths > 8 mm [33].

In the present cohort, functional status at discharge was found to be considerably worse in ELVO patients compared with non-ELVO patients when referenced to the pre-event or pre-admission baseline. This is clinically plausible because the outcome following emergent large vessel occlusion treatment is largely driven by the severity of the index stroke and infarct burden, which limits the likelihood of returning to the pre-event functional level, even when recanalization and lesion stabilization are achieved.

Concurrently, the findings of this study indicate that neurological recovery during hospitalization is a prevalent occurrence in ELVO patients. Improvement was observed across a broad range of baseline NIHSS values, whereas worse outcomes and mortality were observed to cluster in patients with higher initial NIHSS scores. These findings lend support to the interpretation that a significant proportion of ELVO patients experience an improvement in neurological deficit; however, this does not necessarily translate into the regaining of pre-stroke independence by the time of discharge. This emphasizes the distinction between deficit improvement and functional disability.

Conversely, the primary objective of elective stenting in non-ELVO patients is to preserve independence and prevent further deterioration. In accordance with this objective, non-ELVO patients exhibited a reduced frequency of very high NIHSS values, a more prevalent occurrence of stable or improved early outcomes, and a generally less pronounced worsening in discharge mRS. In this context, the timely identification of patients who may benefit from intervention and the prompt decision to treat may be advantageous, as it could help prevent recurrent events or progressive deficits before substantial disability accrues.

These between-group differences should not be interpreted as evidence that the procedure is intrinsically more harmful in the emergent setting but rather as reflecting baseline and pathophysiological differences between cohorts. A significant restriction of the present dataset is the absence of standardized long-term follow-up, including the widely reported 90-day mRS endpoint. This restricts interpretation to discharge outcomes.

However, the risk of ischemic complication after stenting in non-ELVO patients should be taken into consideration in comparison with the known high percentage of patients having further ischemic strokes in the territory of the stenotic artery despite aggressive medical management [34]. Importantly, there was no mortality in this group.

In the present cohort, all fatal cases occurred in emergent ELVO patients treated in the vertebrobasilar circulation, thereby underscoring the high-risk nature of posterior circulation large vessel occlusion with underlying atherosclerotic disease. Contemporary multicenter data on acute basilar artery occlusion demonstrate that rescue stenting following thrombectomy failure can yield improved outcomes when compared with no stenting. However, mortality and severe disability remain prevalent, reflecting the predominant impact of infarct burden and brainstem vulnerability on functional ceiling [35]. Predictors and outcome analyses in vertebrobasilar ICAS-related occlusion further support the notion that this subgroup carries a particularly high-risk profile even under contemporary endovascular management [36]. It is evident that procedural complications are also dependent on circulation. Dissection has been identified as a potential complication associated with bailout angioplasty or stenting, and its incidence has been found to be increased in randomized bailout strategies. These findings provide a rationale for the observed vertebrobasilar dissection pattern in this cohort [37]. In contrast, intracranial hemorrhage in our series occurred exclusively in the anterior circulation. However, this difference was not statistically significant and should be interpreted cautiously given the small event counts and the complex interplay of infarct size, reperfusion, and adjunct antithrombotic therapy. This finding is consistent with the heterogeneity reported in contemporary systematic reviews and meta-analyses [38]. Finally, emerging evidence suggests that an earlier transition to rescue stenting after limited unsuccessful thrombectomy attempts may shorten procedures and improve outcomes, supporting the concept early stenting could be advantageous in selected ICAS-related occlusions [39].

### Limitations

The relatively small sample size is indicative of the overall low incidence of symptomatic high-grade internal carotid artery stenosis (ICAS) requiring percutaneous transluminal angioplasty (PTA) and the restricted indications for intracranial stenting. A further limitation is posed by the single-centre design. Whilst this may result in centre-specific selection and treatment effects, it also ensures a consistent clinical decision pathway, standardized peri-procedural management, and homogeneous imaging and procedural techniques within one institution.

The assessment of the interval between the index event and procedure is limited in this retrospective dataset.

A significant constraint pertains to the absence of systematic functional follow-ups at mid-term and long-term intervals. Specifically, 30-day modified Rankin scale (mRS) data were not available for a sufficient number of non-ELVO patients, and standardized 90-day mRS outcomes could not be reliably assessed in this retrospective dataset. Consequently, functional outcome analyses are restricted to discharge assessments, which limit comparability with the standard 90-day endpoints commonly used in stroke trials. For patients with embolic stroke of undetermined source (ESUS), multicenter registry studies with standardized follow-up time points and recurrent stroke events are indicated. Such studies would facilitate more precise evaluation of the long-term effectiveness and safety of emergent percutaneous transluminal angioplasty (PTAS) in intracranial arterial stenosis (ICAS)-related occlusions.

## 5. Conclusions

Even under restrictive conditions, including use as an ultima ratio therapy in clinically challenging patient groups, PTAS remains a valuable treatment option with reasonable effectiveness. This should be balanced against other treatment options, taking into account the complication rate, which is not negligible. However, in an elective setting, complication rates were lower than anticipated, suggesting a possible role of elective PTAS as a preventive strategy in symptomatic high-grade ICAS when medical therapy fails or when hemodynamic compromise persists. Further multicenter studies with larger sample sizes are needed to better define appropriate indications, optimize procedural strategies, and strengthen the level of evidence.

## Figures and Tables

**Table 1:** Baseline and procedural characteristics of all patients.

	Non-ELVO Group (*n* = 31)	ELVO Group (*n* = 32)	*p*-Value
**Baseline characteristics**			
Age [median]	71 (IQR, 59–73)	64 (IQR, 58–71)	0.239
Female sex [*n*]	8 (25%)	8 (36%)	0.365
History of TIA [*n*]	6 (19%)	4 (13%)	0.457
History of stroke [*n*]	28 (90%)	5 (17%)	**<0.001**
Arterial hypertension [*n*]	30 (97%)	29 (91%)	0.317
Current smoker [*n*]	8 (26%)	7 (22%)	0.714
Diabetes mellitus [*n*]	11 (34%)	12 (38%)	0.721
Hyperlipidemia [*n*]	9 (29%)	7 (22%)	0.514
CHD [n]	6 (19%)	5 (16%)	0.697
Atrial fibrillation [*n*]	4 (13%)	6 (19%)	0.525
**Previous medication**			
Anticoagulation [*n*]	5 (16%)	3 (9%)	0.421
None/mono/dual antiplatelets [*n*]	4/6/21 (13%/19%/68%)	20/9/3 (63%/28%/9%)	**<0.001**
Statins [*n*]	25 (81%)	10 (31%)	**<0.001**
**Stenosis characteristics**			
MCA [*n*]	5 (16%)	12 (38%)	**0.023**
ICA [*n*]	11 (36%)	3 (9%)	
Vertebrobasilar [*n*]	15 (48%)	17 (53%)	
Degree [median]	82 (79–89)	80 (71–90)	0.448
**Clinical characteristics**			
mRS pre stroke [median]	1 (IQR, 0–2)	0 (IQR, 0–1)	**0.001**
NIHSS pre-intervention [median]	4 (IQR, 1–10)	13 (IQR, 5–21)	**<0.001**
**Infarction characteristics**			
Embolic infarction [*n*]	12 (39%)	22 (68%)	0.018
Hemodynamic infarction [*n*]	4 (13%)	0 (0%)	
Mix type: embolic and hemodynamic infarction [*n*]	9 (29%)	5 (16%)	
Perforator infarction [*n*]	6 (19%)	5 (16%)	
**Procedural characteristics**			
Balloon-expandable stents vs. self-expanding [*n*]	27/4 (87%/13%)	14/18 (44%/56%)	**<0.001**
Dilatation before vs. after deployment [*n*]	2/5 (7%/16%)	15/8 (47%/25%)	**<0.001**

ELVO, emergent large vessel occlusion; IQR, interquartile range; TIA, transient ischemic attack; CHD, coronary heart disease; MCA, middle cerebral artery; ICA, internal carotid artery; mRS, modified Rankin scale; NIHSS, National Institutes of Health stroke scale. *p*-values < 0.001 are shown in bold to indicate statistical high significance.

**Table 2:** Outcomes of all patients.

	Non-ELVO Group (n = 31)	ELVO Group (n = 32)	*p*-Value
**Primary outcome**			
Technical success * [*n*]	31 (100%)	32 (100%)	
Reperfusion success ** [*n*]	-	30 (94%)	
Periinterventional complications (dissection, SAH, intracerebral hemorrhage (PH2), and stent thrombosis) [*n*]	0 (0%)	5 (16%)	**0.022**
- Vessel dissection [*n*]	0 (0%)	2 (6%)	0.157
- Subarachnoid hemorrhage [*n*]	0 (0%)	0 (0%)	
- Intracerebral hemorrhage (PH2) [*n*]	0 (0%)	2 (6%)	0.157
- Acute/subacute stent thrombosis [*n*]	0 (0%)	2 (6%)	0.157
**Secondary outcome**			
Neurological improvement [*n*]	13 (42%)	16 (50%)	0.521
Neurological deterioration [*n*]	2 (6%)	7 (22%)	**0.041**
Death (in-hospital) [*n*]	0 (0%)	5 (16%)	**0.022**
mRS at discharge [median]	2 (IQR, 1–3)	4 (IQR, 2–5)	**0.02**
mRS 0–2 at discharge [*n*]	17 (55%)	10 (31%)	0.059

ELVO, emergent large vessel occlusion; PH2, parenchymal hemorrhage type 2 according to the ECASS classification; mRS, modified Rankin scale; IQR, interquartile range. * Technical success defined as a residual stenosis of <50%; ** Reperfusion success defined as complete or almost complete reperfusion (mTICI ≥ 2b). *p*-values < 0.05 are shown in bold to indicate statistical significance.

## Data Availability

The datasets analyzed during the current study are available from the corresponding author upon request.

## Abbreviations

The following abbreviations are used in this manuscript:

ICAD	intracranial atherosclerotic disease
ICAS	intracranial artery stenosis
PTA	percutaneous transluminal angioplasty
ELVO	emergent large vessel occlusion
DSA	digital subtraction angiography
DAPT	dual antiplatelet therapy
